# Geometric working volume of a satellite positive displacement machine

**DOI:** 10.1038/s41598-024-61773-1

**Published:** 2024-05-16

**Authors:** Pawel Sliwinski

**Affiliations:** Faculty of Mechanical Engineering and Ship Technology, Gdansk University of Technology, Gabriela Narutowicza 11/12 Str, 80-233 Gdansk, Poland

**Keywords:** Working volume, Geometric working volume, Non-circular mechanism, Satellite mechanism, Pump, Motor, Satellite, Rotor, Curvature, Positive displacement, Energy efficiency, Discharge machining, Engineering, Mechanical engineering

## Abstract

This article describes a method for determining the geometric working volume of satellite positive displacement machines (pump and motor). The working mechanism of these machines is satellite mechanism consisting of two non-circular gears (rotor and curvature) and circular gears (satellites). Two variants of the satellite mechanism are presented. In the first mechanism, the rolling line of the rotor is a sinusoid "wrapped" around a circle. In the second mechanism, the rolling line of the rotor is a double sinusoid "wrapped" around a circle. A method for calculating the area of the working chamber as a function of the rotor rotation angle is presented, based on mathematical formulae of the rotor, the curvature and the satellite rolling lines. It has been shown that the second variant of the satellite mechanism is advantageously characterised by a larger difference between the maximum area of the working chamber and the minimum area of this chamber. New mathematical formulas have been proposed to calculate the area of the working chamber for any angle of rotation of the shaft (rotor) based on the maximum and minimum values of the area of this chamber. It was thus confirmed that the geometric working volume depends on the maximum and minimum area of a working chamber and on the height of the satellite mechanism. The analyses of the area of the working chamber were carried out both for the mechanism without gears (the area delimited by the rolling lines of the elements of the mechanism) and for the real mechanism with gears. Differences in the values of these fields were also detected.

## Introduction

Every hydraulic positive displacement machine, i.e. a pump and a hydraulic motor, is characterised by the fact that a ceertain amount of fluid flows through its working mechanism during the rotation of the shaft. Therefore, the working mechanism of positive displacement machines is designed in such a way that the process of filling and emptying of working chambers of this mechanism takes place during the rotation of the shaft. The filling and emptying process is only possible if the volume of the working chambers changes cyclically. Therefore, in every positive displacement machine, each working chamber must change its volume from the minimum V_min_ to the maximum V_max_.

**Figure 1:**
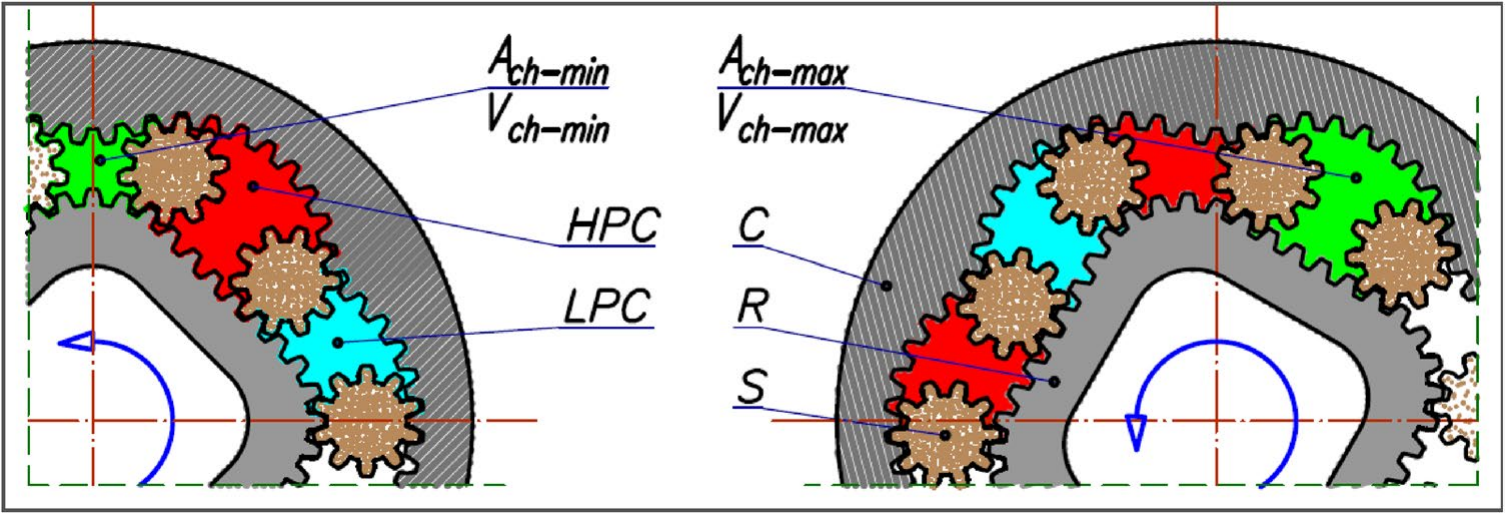
Satellite mechanism type 4 × 6: C curvature, R rotor, S satellite, HPC high-pressure working chamber, LPC low-pressure working chamber ^38,39^.

The sum of the volume changes ΔV_ch_ of all chambers of the working mechanism per one full revolution of the machine shaft is referred to as the geometric working volume q_g_, often called the working volume for short. This is the most important parameter that indicates the size of the displacement machine (pump or hydraulic motor). The unit of geometric working volume is m^3^/rev. In most cases, however, e.g. in catalogues of displacement machines, cm^3^/rev is specified. The geometric working volume can be given in the following form:

1 $${q}_{g}={n}_{ch}\cdot {\Delta V}_{ch}$$

where:

- n_ch_ – the number of cycles (filling and emptying) of the working chambers per one revolution of the shaft;
- ΔV_ch_ – the change in the volume of a working chamber, calculated from the drawing documentation of the working mechanism as:
  2 $${\Delta V}_{ch}={V}_{ch-max}-{V}_{ch-min}$$
- V_k-max_ – the maximum volume of a working chamber (Fig. 1),
- V_k-min_ – the minimum volume of a working chamber (Fig. 1).

The volume of the working chamber V_ch_ and thus the geometric working volume q_g_ of the positive displacement machine is determined on the basis of the design documentation of this machine (as in^1,2^) or on the basis of precise measurements of the elements of the working mechanism. Moreover, the geometric working volume q_g_ is not the theoretical working volume q_t_. In articles^3–5^ it was shown that the geometric working volume q_g_ differs from the theoretical working volume q_t_. It was also shown that it depends on the pressure difference Δp_i_ in the working chambers, i.e. the pressure difference Δp_i_ causes elastic deformation of these chambers and thus an increase in their volume. The so-called actual working volume q_r_ was defined in this way. This volume should be used to calculate the volumetric, mechanical and pressure efficiency. However, neither the geometric working volume q_g_ nor the theoretical working volume q_t_ should be used to calculate these efficiencies^3–5^.

According to^3–6^, the theoretical working volume should be understood as the amount of fluid flowing in the positive displacement machine during one complete revolution without volume losses (and therefore with Δp_i_ = 0) and in the absence of other phenomena affecting this flow. So far, however, a simplification has been used and the theoretical working volume or even the geometric working volume is used to calculate the above-mentioned partial efficiencies, as for example in^7–12^. In this way the geometric working volume, albeit incorrectly, is identified with the theoretical working volume, as for example in^13^. In the literature, for example in^2,14–17^, there is a very general concept, namely specific absorption.

It is desirable that for a given geometric working volume q_g_ the change in the volume ΔV_ch_ of the working chamber is maximal. Then a working mechanism with a lower height H and therefore lower mass is obtained. In this way, a favourably lower mass M of the positive displacement machine is achived.

The subject of this article is the geometric working volume of a positive displacement machine, whose working mechanism is the so-called satellite mechanism (Fig. 1). The structure and operation principle of this mechanism have been described in many publications, including^3–5,18–26^ and the method of its construction in^14,25,26^. It should be remembered that in these mechanisms, the volume V_ch_ of the chamber changes from the V_ch-min_ to the V_ch-max_ during the rotation of the rotor. These volumes are expressed by formulae^6^:

3 $${V}_{ch-max}=H\cdot {A}_{ch-max}$$

4 $${V}_{ch-min}=H\cdot {A}_{ch-min}$$

where:

- H – the height of the mechanism,
- A_ch-max_ – the area of the working chamber with the maximum volume (Fig. 1),
- A_ch-min_ – the area of the working chamber with the minimum volume (Fig. 1).

The change in the volume of the working chamber is therefore:

5 $${\Delta V}_{ch}=H\cdot {\Delta A}_{ch}$$

6 $${\Delta A}_{ch}={A}_{ch-max}-{A}_{ch-min}$$

During one complete rotation of the rotor (360° rotation), the volume of all working chambers changes in the following number of cycles^6,14^:

7 $${n}_{ch}={n}_{C}\cdot {n}_{R}$$

where:

- n_C_ – the number of curvature humps,
- n_R_ – the number of rotor humps.

Therefore, the geometric working volume q_g_ of the satellite mechanism is calculated according to the following formula^3–6^:

8 $${q}_{g}={n}_{ch}\cdot {\Delta V}_{ch}$$

The most important parameters for the calculation of the geometric working volume q_g_ are thus the areas of the working chambers A_ch-max_ and A_ch-min_. The first attempts to calculate these areas were made by Kujawski and Drogosz^2,14–17^. However, they referred a satellite mechanism with a three-hump rotor and a four-hump curvature (3 × 4 type mechanism as in^27–30^). The article^15^ presents general equations for calculating the "specific absorption" of a motor with a 3 × 4 type satellite mechanism:

9 $${q}_{g}=0.5\cdot H\cdot {n}_{C}\cdot {n}_{R}\cdot{\int }_{{\alpha }_{R-min}}^{{\alpha }_{R-max}}\left({r}_{R-1}\cdot {r}_{E-1}-{r}_{R-2}\cdot {r}_{E-2}\right)\cdot {d\alpha }_{R}$$

where:

- α_R_ – the angle of rotation of the rotor;
- α_R-min_ – the angle of rotation of the rotor corresponding to the working chamber with A_ch-min_;
- α_R-max_ – the angle of rotation of the rotor corresponding to the working chamber with A_ch-max_;
- r_R-1_ – the distance from the axis of the rotor to the point of contact of the first satellite with the rotor;
- r_R-2_ – the distance from the axis of the rotor to the point of contact of the second satellite with the rotor (adjacent to the first satellite);
- r_E-1_ – the distance from the axis of the rotor to the point of contact of the first satellite with the curvature;
- r_R-2_ – the distance from the axis of the rotor to the point of contact of the second satellite with the curvature (adjacent to the first satellite).

The above formula is therefore based on the equations of the rolling lines of the rotor and curvature and is therefore valid for a satellite mechanism without teeth. In^16^ a simplified function is presented that describes the rolling line of the rotor of this mechanism (3 × 4) in polar coordinates:

10 $${r}_{R}=a\cdot \left(1-p\cdot {\text{cos}}\left({n}_{R}\cdot {\alpha }_{R}\right)\right)$$

where a and p are constants. The equations for the curvature rolling line were not disclosed. In^16^ an attempt was made to compare the obtained result of the calculations of the change ΔA_ch_ in the field of the working chamber with the results of the planimeter and with the results for the real motor (with a geared mechanism), but he did not explain how he measured the areas of the chambers of the real mechanism. The differences reached almost 3%. However, in^17^ the results of the first calculations of the "specific absorption" of various satellite motors using the AutoCAD programme were presented. In this program, the pitch lines of the mechanism were drawn, a mechanism (without teeth) was created and the A_ch-max_ and A_ch-min_ areas of the working chambers were determined. The issues of areas and "specific absorption" were dealt with in a similar way in^2^. This paper also proposes a mathematical formula for calculating the A_ch_ area of the working chamber as a function of the rotor rotation angle α_R_. This formula is described in Sect. 6.1.

In ^26^ it was proposed to calculate the area of the working chamber of a 4 × 6 satellite mechanism for any rotor rotation angle α_R_. However, this is a very general proposal, as the authors presented a mathematical formula to describe the rotor pitch line but did not provide a formula to calculate the pitch line of the curvature (similarly as in^16^). Therefore, it is not possible to calculate the area under the pitch line of the curvature in a simple analytical way. It is also not specified according to which function the value of the working chamber changes depending on the rotor rotation angle α_R_ from A_ch-min_ to A_ch-max_. However, they suggest calculating the working volume of the 4 × 6 type satellite mechanism using the following empirical formula (approximate formula)^26^:

11 $${q}_{g}=\frac{1}{20}\cdot H\cdot {m}^{2}\cdot {\left(\frac{{z}_{R}}{{n}_{R}}-{z}_{S}\right)}^{2}\cdot \left(1.433\cdot {\left(\frac{{n}_{R}\cdot {z}_{S}}{{z}_{R}-{n}_{R}\cdot {z}_{S}}\right)}^{1.481}+2.144\right)$$

where:

- m—The tooth modulus,
- z_S_—The number of teeth on the satellite,
- z_R_—The number of teeth on the rotor.

The above formula only applies to only for the 4 × 6 satellite mechanism, in which the pitch line of the rotor is described by the high-order ellipses. Therefore, the above formula does not work for mechanisms with a different rotor curve.

The methods previously known in the literature for calculating the working volume of a satellite machine referred to the calculation of this volume in a 3 × 4 type satellite mechanism. Furthermore, these methods were based on the determination of the A_ch-max_ and A_ch-min_ areas of the working chambers, which are generated by the pitch lines of the rotor, the curvature and the satellites. Therefore, the areas for the real mechanism, i.e. the toothed mechanism, were not calculated.

Therefore, there is no information in the literature about the differences in the values of the A_ch-max_ and A_ch-min_ areas of the working chambers of toothed and non-toothed mechanisms and how the number of teeth in the mechanism affects the above-mentioned areas. Furthermore, the publication ^2^ does not state whether the proposed mathematical formula for calculating the working chamber area A_ch_ as a function of the rotor rotation angle α_R_ is suitable for calculating the chamber area of a real (i.e. toothed) mechanism and, if so, with what error. The aim of this article is therefore to dispel the above-mentioned doubts and to propose a new mathematical formula for calculating the area A_ch_ of the working chamber as a function of the rotor rotation angle α_R_. Positive displacement machines with a 3 × 4 satellite mechanism are no longer manufactured (mainly because of the small number of working chambers and the hydrostatic imbalance (as a result of the pressure in the working chambers, the shaft is bent and the bearings are heavily loaded)). This article therefore analyses the latest 4 × 6 satellite mechanisms.

## New construction of satellite mechanisms

**Figure 2:**
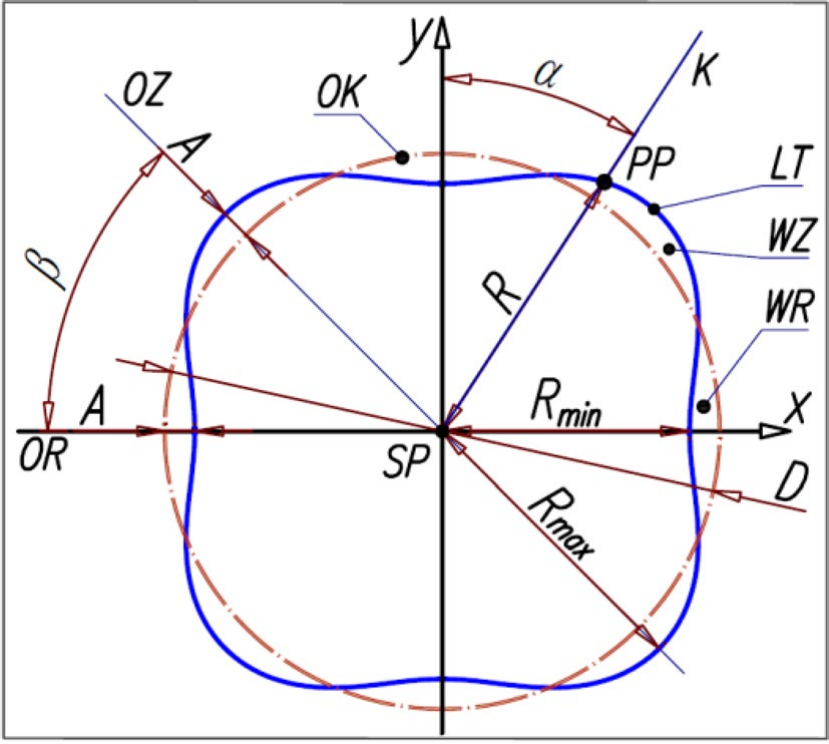
Parameters of the rotor pitch line of the 4 × 6 type satellite mechanism – variant I: K the half-line with the origin in the centre SP of the rotor rotation and passig through the point PP on the pitch line LT, WZ – the hump convexity, WR the hump concavity, OK circle with diameter D, OZ the axis of symmetry of the hump convexity, OR the axis of symmetry of the hump concavity, PP point of intersection of the half-line K with the pitch line LT, β —The angle between the axis OR and the axis OZ. Other designations in the tekst ^22^.

According to the methodology described in^14^, two variants of the 4 × 6 satellite mechanism were developed, i.e.:

1) first variant (I) (Fig. 2) – the rotor pitch line is described by the following equation^22^:

12 $$R=\frac{D}{2}-A\cdot {\text{cos}}\left({n}_{R}\cdot \alpha \right)$$

**Figure 3:**
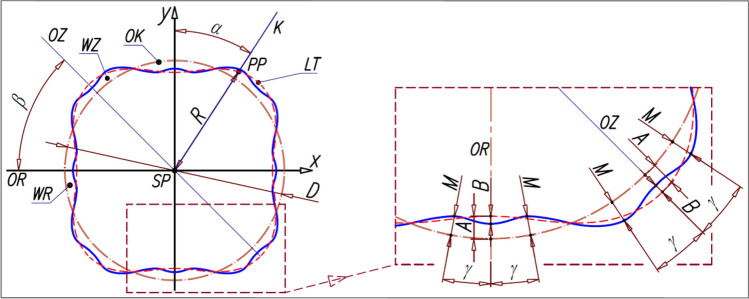
Parameters of the rotor pitch line of the 4 × 6 type satellite mechanism—variant II. Designations as in Fig. 2 and in the text ^23^.

2) second variant (II) (Fig. 3) – the rotor pitch line is described by^23^:

13 $$R=\frac{D}{2}-A\cdot \mathit{cos}\left({n}_{R}\cdot \alpha \right)+B\cdot \mathit{cos}\left(2\cdot {n}_{R}\cdot \alpha \right)$$

where (Fig. 2 and Fig. 3):

D – the diameter of the base circle OK of the rotor,

α – the angle with the origin in the centre SP of the rotor rotation and with the arms formed by any axis of the XY coordinate system and the line K,

A – the distance of the point PP from the circle OK for B = 0, which lies in the axis of symmetry OZ of the hump convexity WZ and in the axis of symmetry OR of the hump concavity WR,

B – the amount by which the distance of the point P from the circle OK is reduced.

If $B\ge \frac{A}{4}$, then the maximum distance M of the point PP from the circle OK is to the left and right of the axis OZ of the hump convexity WZ and to the left and right of the axis OR of the hump concavity WR, at the distance of the angle γ from these axes, where^23^:

14 $$\gamma =\frac{1}{{n}_{R}}\cdot {\text{arccos}}\left(\frac{A}{{n}_{R}\cdot B}\right)$$

If $B<\frac{A}{4}$, then the maximum distance M of the point PP from the circle OK lies in the axis of symmetry OZ of the hump convexity WZ and in the axis of symmetry OR of the hump concavity WR and for the hump convexity WZ is^23^:

15 $$M=R-\frac{D}{2}$$

However, for the hump concavity WR is^23^:

16 $$M=\frac{D}{2}-R$$

The large pressure difference acting on the satellite causes high pressures in the area of the interacting teeth of the mechanism elements^20^ and high bending stresses in the tooth root. It is therefore recommended to use as few teeth as possible on the satellite, which makes the tooth thicker. In typical gears, the minimum limit number of teeth is 17, below which undercutting of the tooth root occurs^31^. If slight undercutting is allowed, the minimum limit number of teeth can be 14^31^. To increase the durability of the teeth of the satellite mechanism, it was decided to use satellites with a very low number of teeth, i.e. 10 or less^14,21–23,32,33^. For example:

- In ^21^ and in a satellite mechanism is presented in which the satellite has 10 teeth;
- In ^22,23,32^ a satellite mechanism is presented in which the satellite has 9 teeth;
- In ^14^ a satellite mechanism is presented in which the satellite has 8 teeth;
- In ^33^ a satellite mechanism is presented in which the satellite has 5 teeth and these are teeth with a circular-arch shape.

Then, in order to avoid undercutting the tooth root, both the head and the root are low. This means that both the height h_a_ of the tooth head and the height h_f_ of the tooth root are smaller than the tooth modulus m (Table 1).

**Table 1:** Basic parameters of the satellite mechanisms with the nine-toothed satellite.

parameters common to variants I and II				
n_R_	n_E_	z_s_	z_R_	z_E_
4	6	9	40	60
D_p-S_	D_r-S_	Α_p-S_	H_a-S_	H_f-S_
9 mm	8.84816 mm	30^o^	0.855 mm	0.900 mm
m	β	h_a-CH_	h_f-CH_	–
1.0 mm	45^o^	0.900 mm	0.857 mm	–
correction coef. of the satellite and chisel teeth			Δh_a_	Δh_f_
x = – 0.07592			0.045	0.043 mm
parameters for mechanism of variant I				
A	D	–	–	–
1.6663 mm	38.8795 mm	–	–	–
parameters for mechanism of variant II				
A	B	D	M	γ
2.3478 mm	0.41086 mm	37.5648 mm	1.937 mm	–
Areas of the satellite S and chisel CH (in mm^2^) (Fig. 6)				
	A_a_	A_f_	A_a-S-CH_	A_f-S-CH_
Satellite S	0.9226	0.9395	0.0182	0.0258
Chisel CH	0.9408	0.9137		

The subscript “CH” refers to the chisel.

## Considered satellite mechanisms

**Figure 4:**
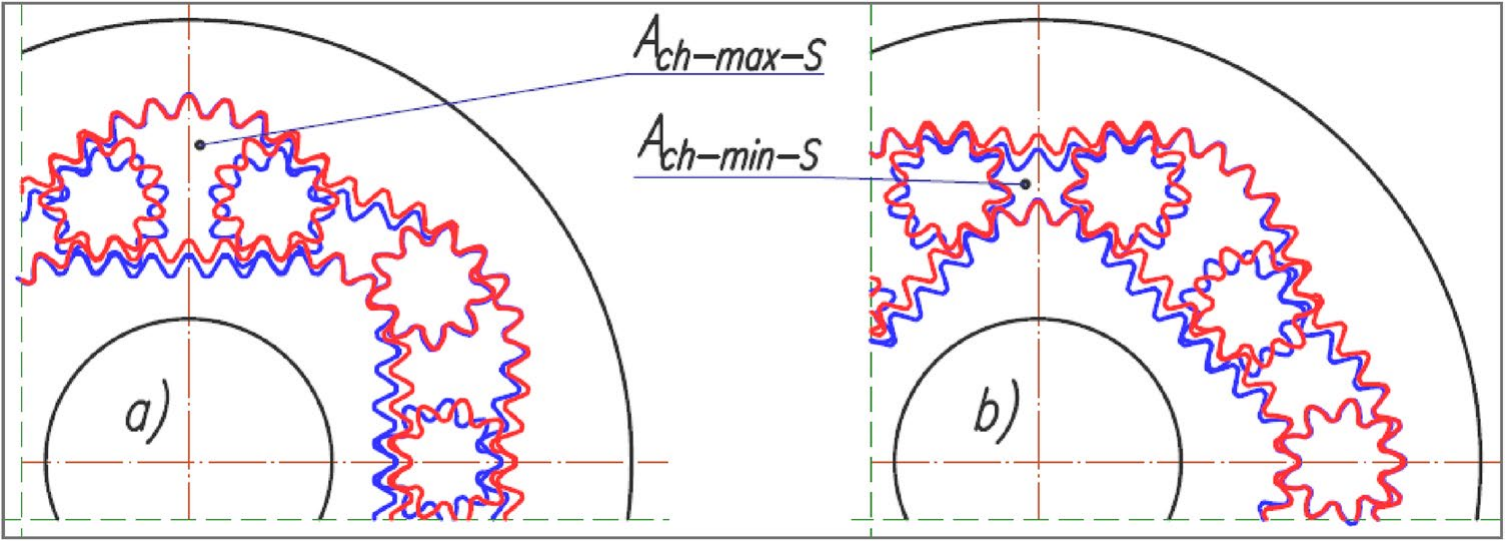
Satellite mechanism with nine-toothed satellite—variant I (red colour) and variant II (blue colour): (**a**) comparison of the maximum area A_ch-max_ of the working chamber of these mechanisms; (**b**) comparison of the minimum area A_ch-min_ of the working chamber of these mechanisms.

**Figure 5:**
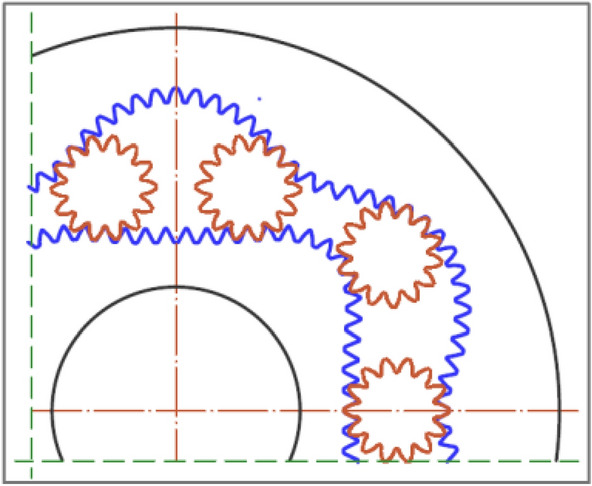
Satellite mechanism variant II with fourteen-toothed satellite.

**Figure 6:**
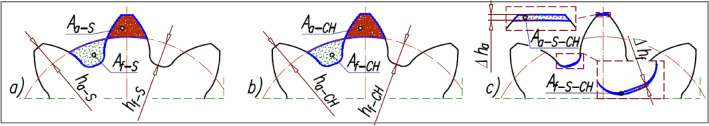
Areas of the satellite (**a**), the chisel (**b**) and the difference between the areas of the chisel and the satellite (**c**).

In this article, satellite mechanisms according to the patent descriptions^22,23^ are considered, where the number of satellite teeth is 9 and 14 (Fig. 4 and Fig. 5). The parameters of these mechanisms are listed in Table 1, Table 2 and Fig. 6. When designing these mechanisms, the aim was to maximise the values of parameters A and B (Table 1) while complying with the conditions described in^14^ in order to obtain the largest possible geometric working volume q_g_.

## Area of the working chamber

### Methods for determining the area of the working chamber

**Figure 7:**
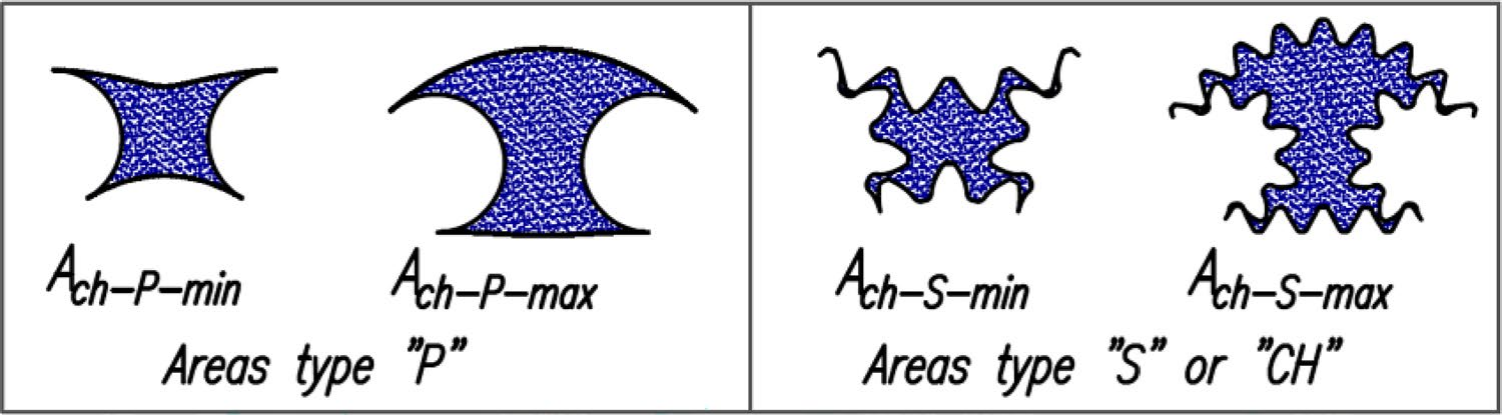
Minimum and maximum areas of the working chamber determined from the CAD documentation on the basis on the pitch lines of the mechanism elements and the real areas (toothed elements).

Figure 1 and Fig. 4 show the location of the satellite mechanismu for which the minimum A_min_ and the maximum A_max_ area of the working chamber are determined (Figs. 6 and 7 shows the minimum and maximum areas of the working chamber, which were determined using the CAD documentation based on the pitch lines of the mechanism elements (A_ch-P-min_ and A_ch-P-max_) and the real areas (toothed elements – (A_ch-S-min_ and A_ch-S-max_).

The area A_ch_ of the working chamber for any angle α_R_ of the rotor rotation can be determined in two ways, namely:

1. by direct reading from the documentation of the working mechanism in the CAD programme;
2. analytical method – by setting up the mathematical formula A_ch_ = f(R,α_R_).

The first method is very easy and quick to use, and all the areas of the working chambers were calculated in this way.

The second method requires knowledge of mathematical functions that describe the edges of the area. In the case of real areas of working chambers, i.e. toothed areas, finding such functions is very difficult (due to the complicated geometry of the teeth and their orientation in the coordinate system). For this reason, the analytical method can be limited to determining the areas of the working chambers, the edges of which are the pitch lines of the rotor teeth, the curvature teeth and satellites teeth. The pitch line of the rotor is described by the formula (12) or (13). However, the pitch line of the curvature is determined by a series of points (x_E_,y_E_), whose values are calculated according to the method described in^19^. Therefore, the pitch line of the curvature cannot be described by a simple mathematical formula.

The area of the working chamber is:

17 $${A}_{ch}={A}_{C}-{A}_{R}-{A}_{S1}-{A}_{S2}$$

where:

18 $${A}_{C}=\frac{1}{2}\cdot {\int }_{{\varphi }_{1}}^{{\varphi }_{2}}{\left({f}_{C}\right)}^{2}d\varphi =\frac{1}{2}\cdot {\sum }_{i=1}^{n}\left({{R}_{E}}^{(i)}\cdot {{\Delta L}_{E}}^{(i)}\right)$$

A_C_ – the area bounded by the pitch line of the curvature (between points E^(1)^ and E^(2)^):

R_E_^(i)^ – the distance from the centre of the mechanism to point E^(i)^ on the curvature (the radius of the curvature);

ΔL_E_^(i)^ – the length of the „i”-th elementary section of the curvature pitch line, calculated according to the method described in^14^;

n – the number of elementary sections of the curvature pitch line between the points E^(1)^ and E^(2)^;

A_R_ – the area bounded by the rotor pitch line (between the points F^(1)^ and F^(2)^):

19 $$A_{C} = \frac{1}{2}\,\mathop \smallint \limits_{{\varphi_{1} }}^{\varphi } \left( {f_{R} } \right)^{2} d\varphi$$

20 $$f_{R} = \frac{D}{2} - A\,\cos \left( {n_{R} \,\left( {\alpha + \alpha_{R} } \right)} \right)$$

or

21 $${f}_{R}=\frac{D}{2}-A\cdot {\text{cos}}\left({{\text{n}}}_{{\text{R}}}\cdot \left(\mathrm{\alpha }+{\alpha }_{R}\right)\right)+B\cdot {\text{cos}}\left(2\cdot {{\text{n}}}_{{\text{R}}}\cdot \left(\mathrm{\alpha }+{\alpha }_{R}\right)\right)$$

f_R_—The function that describes the rotor pitch line after it has been rotated by an angle α_R_:

A_S1_—The area of a fragment of a satellite centred on the point S^(1)^;

κ_1_—The central angle of a satellite centred on the point S^(1)^ in deg;

A_S2_—The area of a fragment of a satellite centred on the point S^(2)^:

κ_2_—The central angle of a satellite centred on the point S^(2)^ in deg.

22 $$A_{S1} = \frac{{D_{p - S}^{2} }}{4}\,\left( {1 - \frac{{\kappa_{1} }}{360} + \frac{{\sin \kappa_{1} }}{2}} \right)$$

23 $$A_{S} 2 = D_{{(p - S)^{2} }} /4\,(1 - \kappa_{2} /360 + \sin \,\kappa_{2} /2)$$

In^2^ and^26^ it was shown that the area of the working chamber of the satellite mechanism is directly proportional to the square of the tooth modulus. It is obvious that the pitch diameter of the satellite is also proportional to the number of teeth. Therefore, we can assume that the following relationship is true:

24 $${A}_{ch}={C}_{ch}\cdot {m}^{2}\cdot {{z}_{S}}^{2}$$

where C_ch_ is a proportionality coefficient that depends on the type of satellite mechanism and the geometrical parameters of this mechanism (as in Table 1 and Table 2). This coefficient also depends on whether we consider a non-toothed or a toothed chamber. Moreover, this coefficient takes values from C_ch-min_ to C_ch-max_ for a chamber whose area varies between A_ch-min_ and A_ch-max_.

**Table 2:** Basic parameters of the satellite mechanisms variant II with the fourteen-toothed satellite.

n_R_	n_E_	z_S_	z_R_	z_E_
4	6	14	64	96
D_p-s_	D_r-s_	α_p-S_	H_a-s_	H_f-s_
9 mm	9.10 mm	30^o^	0.5496 mm	0.810 mm
m	x	β	H_a-CH_	H_f-CH_
0.643 mm	0.0785	45^o^	0.5786 mm	0.7662 mm
Correction coef. of the satellite and chisel teeth			Δh_a_	Δh_f_
x = 0.0785			0.029	0.0438 mm
Areas of the satellite S and chisel CH (in mm^2^) (Fig. 6)				
	A_a_	A_f_	A_a-s-CH_	A_f-S-CH_
Satellite S	0.3805	0.4882	0.0084	0.0184
Chisel CH	0.3889	0.4698		

The subscript “CH” refers to the chisel.

### The values of the areas of the working chamber

**Figure 8:**
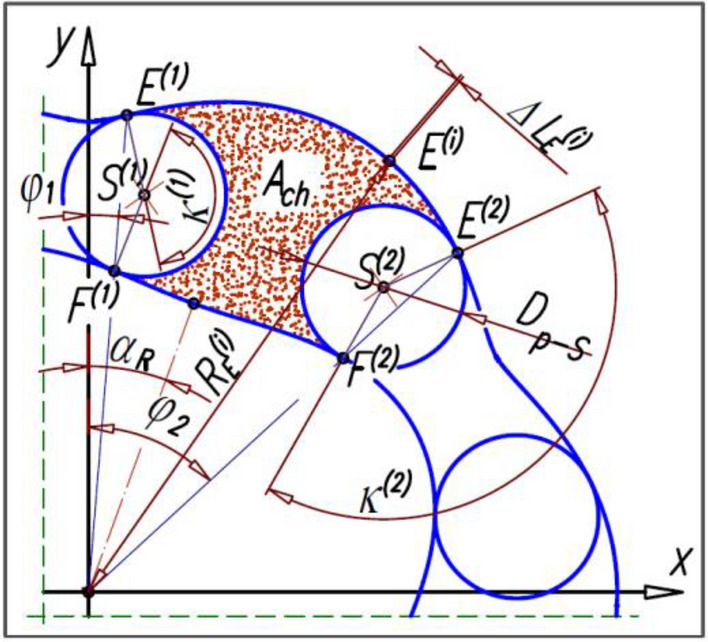
Area A_ch_ of the working chamber with characteristic points for each angle α_R_ of the rotor rotation.

Table 3 shows the minimum and maximum values of the working chamber area for both variants of the satellite mechanism and for:

- the real (toothed) satellite mechanism – A_ch-S-min_ and A_ch-S-max_ areas;
- the backlash-free mechanism (that is the satellite is a chisel – as in Fig. 6b) – A_ch-CH-min_ and A_ch-CH-max_ areas;
- the areas determined by the pitch line of the rotor, the curvature and the satellites – A_ch-P-min_ and A_ch-P-max_ areas;
- the number of teeth on the satellite 9 and 14 respectively – in variant II of the mechanism (Fig. 8).

**Table 3:** Minimum and maximum area of the working chamber in mm2.

		A_ch-P-min_	A_ch-P-max_	A_ch-S-min_	A_ch-S-max_	A_ch-CH-min_	A_ch-CH-max_
Variant I		48.7266	108.9258	48.3736	108.3422	47.9652	107.9052
Variant II	9 teeth	32.7636	120.5814	33.2226	121.0698	32.9421	120.6625
	14 teeth			33.4174	121.6259	33.4001	121.3398
δA_ch-I-II_	9 teeth	–32.76%	10.70%	–31.32%	11.75%	–31.32%	11.75%
	14 teeth			–30.92%	12.26%	–30.36%	12.45%
δA_ch-9–14_		–	–	0.58%	0.46%	1.39%	0.56%

Table 3 are also shows the relative differences in the areas of the working chamber for variants I and II of the mechanism, calculated as:

25 $${\delta A}_{ch-I-II}=\left(\frac{{A}_{ch-II}}{{A}_{ch-I}}-1\right)\cdot 100$$

and the relative differences in the areas of the working chambers in variant II of the mechanism for a satellite with 9 and 14 teeth and calculated as:

26 $${\delta A}_{ch-9-14}=\left(\frac{{A}_{ch-14}}{{A}_{ch-9}}-1\right)\cdot 100$$

Table 4 Shows the relative differences in the above-mentioned areas, calculated as follows:

**Table 4:** Relative differences (in %) of the areas Ach-max and Ach-min of the working chamber.

		δA_ch-P-S-min_	δA_ch-P-S-max_	δA_ch-P–CH-min_	δA_ch-P-CHmax_	δA_ch-S-CH-min_	δA_ch-S-CH-max_
Variant I		− 0.73	− 0.54	− 1.56	− 0.94	− 0.85	− 0.40
Variant II	9 teeth	1.40	0.40	0.54	0.07	− 0.85	− 0.34
	14 teeth	2.0	0.87	–	–	–	–

27 $${\delta A}_{ch-P-S}=\left(\frac{{A}_{ch-S}}{{A}_{ch-P}}-1\right)\cdot 100$$

28 $${\delta A}_{ch-P-CH}=\left(\frac{{A}_{ch-CH}}{{A}_{ch-P}}-1\right)\cdot 100$$

29 $${\delta A}_{ch-S-CH}=\left(\frac{{A}_{ch-CH}}{{A}_{ch-S}}-1\right)\cdot 100$$

30 $$\delta \left({\Delta A}_{ch-P-I-II}\right)=\left(\frac{{\Delta A}_{ch-P-II}}{{\Delta A}_{ch-P-I}}-1\right)\cdot 100$$

31 $$\delta \left({\Delta A}_{ch-S-I-II}\right)=\left(\frac{{\Delta A}_{ch-S-II}}{{\Delta A}_{ch-S-I}}-1\right)\cdot 100$$

32 $$\delta \left({\Delta A}_{ch-CH-I-II}\right)=\left(\frac{{\Delta A}_{ch-CH-II}}{{\Delta A}_{ch-CH-I}}-1\right)\cdot 100$$

33 $$\delta \left({\Delta A}_{ch-P-S}\right)=\left(\frac{{\Delta A}_{ch-S}}{{\Delta A}_{ch-P}}-1\right)\cdot 100$$

34 $$\delta \left({\Delta A}_{ch-P-CH}\right)=\left(\frac{{\Delta A}_{ch-CH}}{{\Delta A}_{ch-P}}-1\right)\cdot 100$$

35 $$\delta \left({\Delta A}_{ch-S-CH}\right)=\left(\frac{{\Delta A}_{ch-CH}}{{\Delta A}_{ch-S}}-1\right)\cdot 100$$

**Figure 9:**
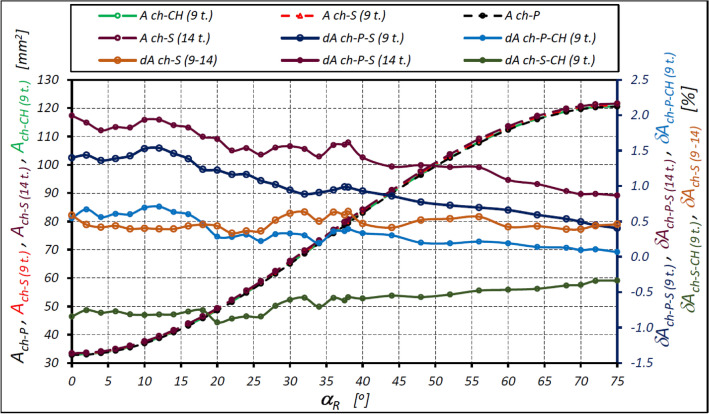
Areas A_ch_ of the working chamber as a function of the angle α_R_ of the rotor rotation: A_ch-P_ – the area created by the pitch line of the rotor, the curvature and the satellites; A_ch-S (9 t.)_ – the area of the toothed chamber for z_S_ = 9; A_ch-S (14 t.)_ – the area of the toothed chamber for z_S_ = 14; A_ch-CH (9 t.)_ – the area of the toothed chamber for z_S_ = 9 (backlash-free mechanism); δA_ch-P-S (9 t.)_ – the relative difference between the areas of A_ch-P_ and A_ch-S (9 t.)_; δA_ch-P-S (14 t.)_ – the relative difference between the areas of A_ch-P_ and A_ch-S (14 t.)_; δA_ch-P–CH (9 t.)_ – the relative difference between the areas of A_ch-P_ and A_ch-CH (9 t.)_; δA_ch-S-CH (9 t.)_ – the relative difference between the areas of A_ch-S_ and A_ch-CH (9 t.)_; δA_ch-S (9–14)_ – the relative difference between the areas of A_ch-S (9 t.)_ and A_ch-S (14 t.)_.

Figure 9 shows the characteristics of the areas of the working chamber of the mechanism of variant II and the relative differences depending on the angle α_R_ of rotation of the rotor for all the option mentioned above, i.e. according to the pitch line, the real mechanism (toothed) and the backlash-free mechanism (satellite = chisel) (Table 5).

**Table 5:** Differences of the maximum and minimum areas (in mm2) and their relative differences (in %).

		ΔA_ch-P_	ΔA_ch-S_	ΔA_ch-CH_	Δ(ΔA_ch-P-S_)	Δ(ΔA_ch-P–CH_)	Δ(ΔA_ch-S-CH_)
Variant I		60.1992	59.9686	59.940	–0.38%	− 0.43%	–0.05%
Variant II	9 teeth	87.8178	87.8472	87.7204	0.03%	− 0.11%	–0.14%
	14 teeth		88.2085	–	0.44%	–	–
δ(A_ch-I-II_)	9 teeth	45.87%	46.49%	46.35%	–	–	–
	14 teeth		47.22%	–	–	–	–

## The comparative index of a positive displacement machine

### The power density

Some scientists, dealers and manufacturers of hydraulic positive displacement machines use the term "power density" for comparative and marketing purposes. This “power density” ρ_P_ is understood as the ratio of the nominal mechanical power P_M_ of the positive displacement machine to its mass M_M_, i.e.^34–37^:

36 $${\rho }_{P}=\frac{{P}_{M}}{{M}_{M}}$$

However, in ^26^ the power density ρ_P_ for satellite machines was defined as:

37 $${\rho }_{P}=\frac{{q}_{g}}{\pi \cdot {{R}_{Cmax}}^{2}\cdot H}$$

where R_Cmax_ is the maximum value of the radius of the pitch line of the curvature.

The above definitions are characterised by a certain imprecision. In the case of formula (36), the mechanical power P_M_ of the machine is a function of the speed of its shaft. Therefore, it is difficult to compare different types of positive displacement machines, because with the same nominal power, the different machines have different nominal rotational speed and different nominal torque. However, formula (37) only indicates the geometric working volume q_g_ that can be accommodated in a cylinder of radius R_Cmax_ and height H. There is therefore no indication of the total mass M_M_ of the device.

### Specific mass of the positive displacement machine

In the context of the inaccuracies described above, a new comparative index for positive displacement machines is proposed, which is defined as follows:

38 $${\rho }_{q}=\frac{{M}_{M}}{{q}_{g}}$$

This index is proposed to denote the specific mass of the positive displacement machine. Logic dictates that the value of this indicator should be compared for machines with the same or similar nominal working pressure p_nom_.

## Models of the working chamber area as a function of the rotor rotation angle

### The know model of the working chamber area

According to^2^ the area A_ch_ of the working chamber of a satellite mechanism changes as a function of the rotor rotation angle α_R_ according to the following relationship:

39 $${A}_{ch}=0.5\cdot \left({A}_{ch-max}-{A}_{ch-min}\right)\cdot \left(1-{\text{cos}}\left({\alpha }_{R}\cdot {n}_{CR}\right)\right)+{A}_{ch-min}$$

where:

40 $${n}_{CR}=\frac{{n}_{C}\cdot {n}_{R}}{{n}_{C}+{n}_{R}}$$

**Figure 10:**
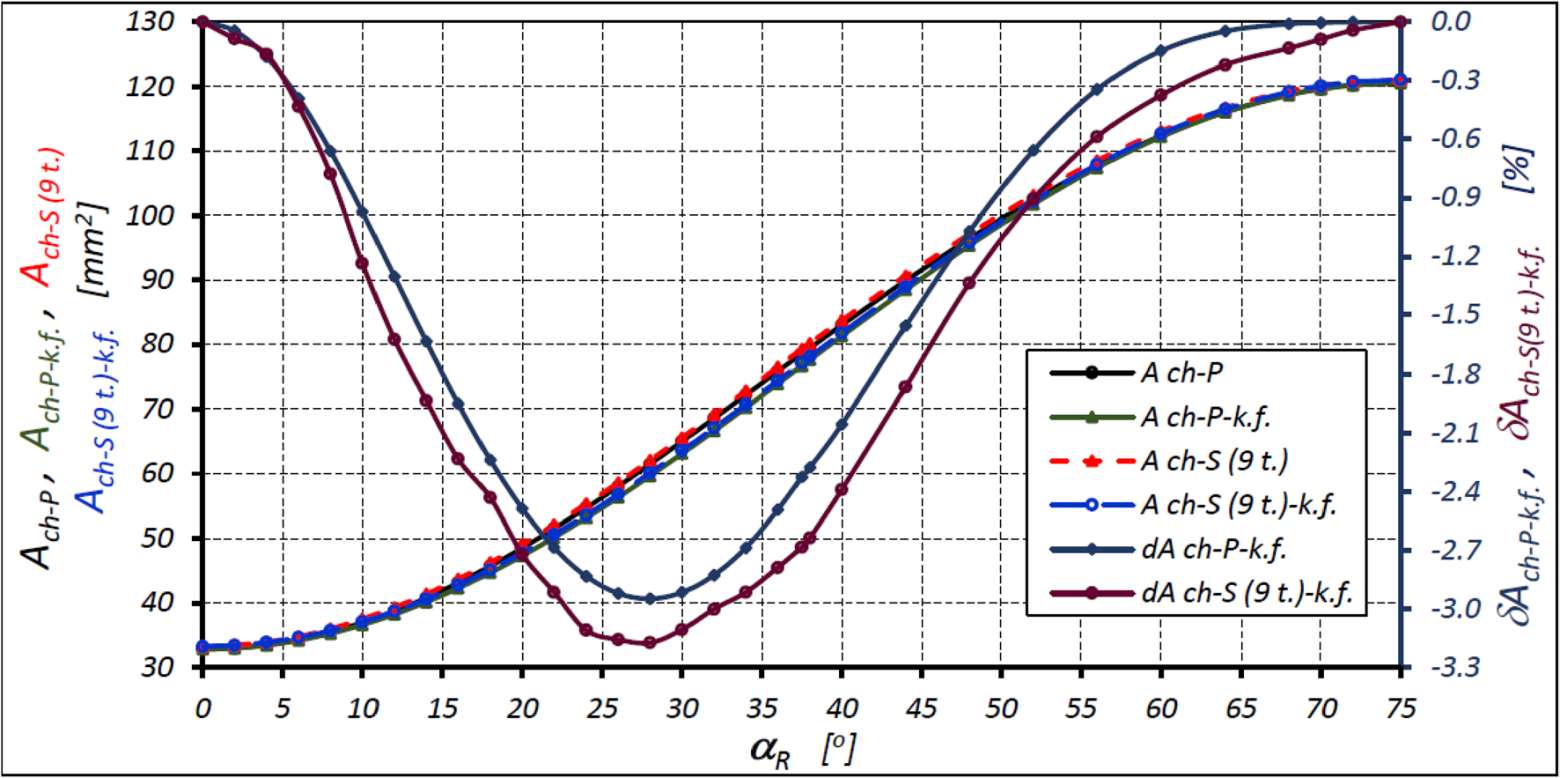
Areas A_ch_ of the working chamber of the satellite mechanism of variant II with the nine-tooth satellite (z_S_ = 9) as a function of the angle α_R_ of the rotor rotation calculated according to the known formula (39) and the relative differences of these areas: A_ch-P_ – the area of the working chamber determined by the pitch line of the rotor, the curvature and the satellites; A_ch-S (9 t.)_ – the area of the toothed chamber; A_ch-P-k.f._ – calculated area of the working chamber A_ch-P_; A_ch-S (9 t.)-k.f._ – calculated area of the working chamber A_ch-S (9 t.)_; δA_ch-P-k.f._ – the relative difference between the areas A_ch-P_ and A_ch-P-k.f._; δA_ch-S(9 t.)-k.f._ – the relative difference between the areas of the areas A_ch-S (9 t.)_ and A_ch-S(9 t.)-k.f._.

Figure 10 shows the characteristics of the areas of the working chamber of the mechanism of variant II (z_S_ = 9) as a function of the rotor rotration angle α_R_. They were determined from the CAD documentation of the mechanism and calculated according to the known formula (39). The relative differences between the values of these areas are also shown in Fig. 10 and are calculated as:

41 $${\delta A}_{ch-k.f}=\left(\frac{{A}_{ch-k.f}}{{A}_{ch}}-1\right)\cdot 100$$

The above formula is a general formula for all considered areas (as in Fig. 7). So, for example, for the area “P” type, instead of A_ch_ will be A_ch-P_ and instead of A_ch-k.f._ will be A_ch-P-k.f._ e.t.c.

The above characteristics show that the use of formula (39) causes an underestimation of the value of the chamber volume by up to 3.3% in the case of a toothed chamber and 3% in the case of a non-toothed chamber. Therefore, it is advisable to look for new formulas that give a smaller error.

### Proposed models for the working chamber area

#### Model I

The differences in the values for the working chamber area resulting from formula (39) can be reduced by extending this formula to the following form:

42 $${A}_{ch-n.f.I}=0.5\cdot \left({A}_{ch-max}-{A}_{ch-min}\right)\cdot \left[1-{\text{cos}}\left({\alpha }_{R}\cdot {n}_{CR}\right)+{\Theta }_{I}\cdot {\text{sin}}\left({\alpha }_{R}\cdot {n}_{CR}\right)\right]+{A}_{ch-min}$$

Where θ_I_ is a coefficient whose value is chosen so that:

43 $$\left|{\delta A}_{ch-n.f.I-max}\right|+\left|{\delta A}_{ch-n.f.I-min}\right|=min.$$

44 $$\left|{\delta A}_{ch-n.f.I-max}\right|\le \left|{\delta A}_{ch-n.f.I-min}\right|$$

In Table 6, the values of the coefficient and the relative differences in the working chamber area of the considered mechanism of variant II are given and calculated as follows:

**Table 6:** The relative differences in % in the calculation of the chamber area of the satellite mechanism of variant II according to model I and values of the coefficient θ_I_.

Mechanism		${\delta A}_{ch-n.f.I-max}$	${\delta A}_{ch-n.f.I-min}$	θ_I_
Type “P” (non-toothed)		0.728%	− 0.728%	0.03408
z_S_ = 9	type “S”	0.741%	− 0.741%	0.03734
	type “CH”	0.702%	− 0.702%	0.03614
z_S_ = 14		0.878%	− 0.878%	0.03679

45 $${\delta A}_{ch-n.f.I}=\left(\frac{{A}_{ch-n.f.I}}{{A}_{ch}}-1\right)\cdot 100$$

The above formula is a general formula for all considered areas (as in Fig. 7). For example, the value A_ch-P_ is used instead of A_ch_ and the value A_ch-P-n.f.I_ is used instead of A_ch-n.f.I_ for the area of type “P” e.t.c.

**Figure 11:**
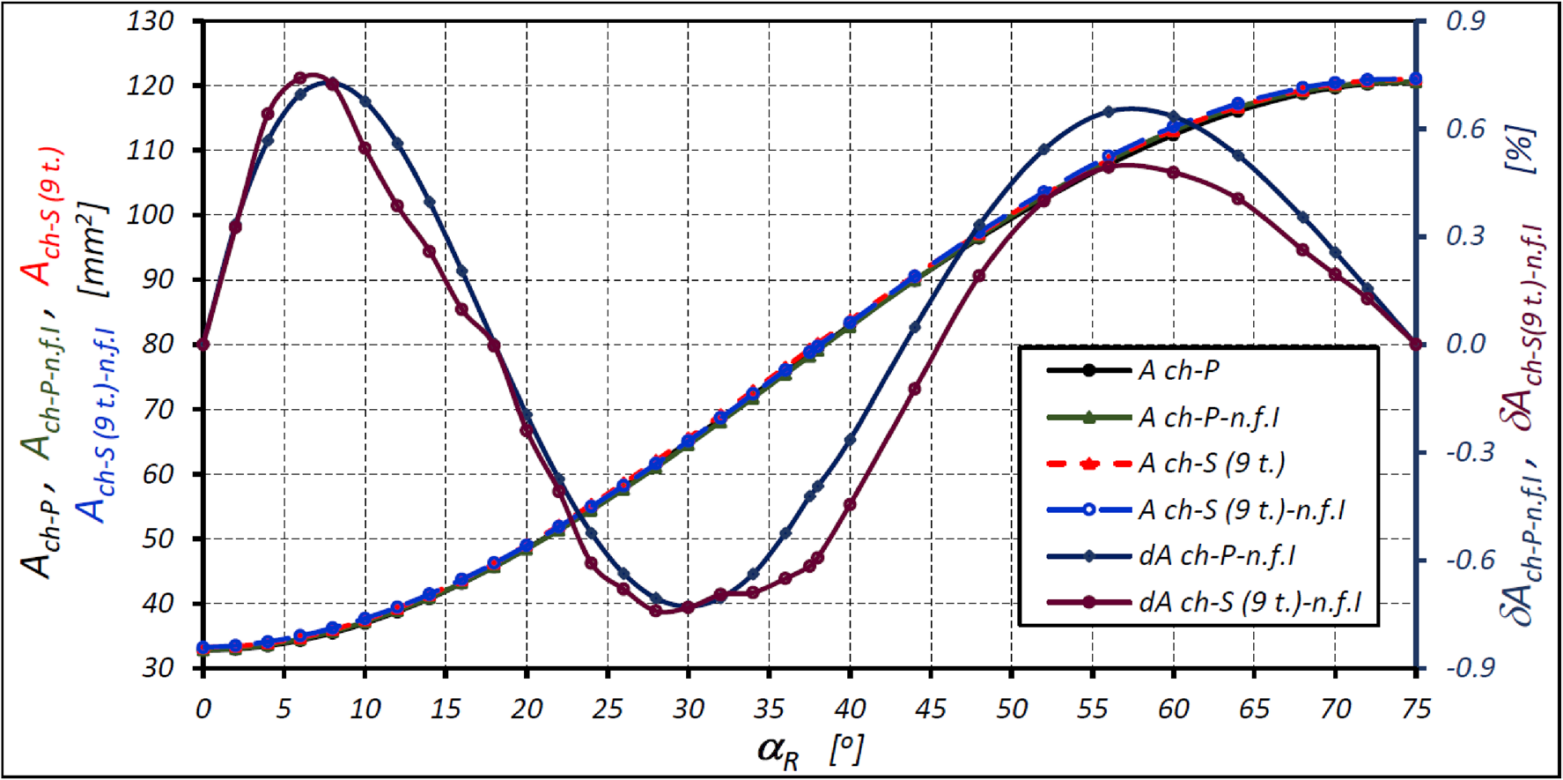
Areas A_ch_ of the working chamber of the satellite mechanism of variant II with nine-tooth satellite (z_S_ = 9) as a function of the angle α_R_ of the rotor rotation calculated according to formula (42) and the relative differences of the areas. Designations as in Fig. 10.

The characteristics of the areas calculated according to formula (42) for the satellite mechanism of variant II with the nine-tooth satellite are shown in Fig. 11.

The above characteristics show that the use of formula (42) means that the relative difference in the calculated area of the working chamber of the mechanism with a nine-tooth satellite is not more than 0.74%. A larger difference was determined for the mechanism with a fourteen-tooth satellite (0.88%—Table 6). However, for α_R_ = (0,18°) and for α_R_ = (45°,75°) the calculated value of the chamber area is greater than the real value of this area. Furthermore, in the ranga α_R_ = (18°,45°), the calculated value of the chamber area is smaller than the actual value of this area.

#### Model II

The differences in the values of the working chamber area resulting from formula (42) can be reduced by expanding this formula into the following form:

46 $${A}_{ch}=0.5\cdot \left({A}_{ch-max}-{A}_{ch-min}\right)\cdot \left[1-{\text{cos}}\left({\alpha }_{R}\cdot {n}_{CR}\right)+{\Theta }_{I}\cdot {\text{sin}}\left({\alpha }_{R}\cdot {n}_{CR}\right)-{\Theta }_{II}\cdot {\text{sin}}\left(2\cdot {\alpha }_{R}\cdot {n}_{R}\right)\right]+{A}_{ch-min}$$

Where θ_II_ is a coefficient whose value is chosen so that conditions (43) and (44) are met.

In Table 7, the values of the coefficient Θ_II_ and the relative differences in the working chamber area of the considered mechanism of variant II are given and calculated according to formula (45). The working chamber areas were calculated according to formula (46) and the CAD documentation (as in Fig. 7).

**Table 7:** The relative differences in % in the calculation of the chamber area of the satellite mechanism of variant II according to model II and the values of the coefficient Θ_II_. Coefficient Θ_I_ as in Table 6.

Mechanism		${\delta A}_{ch-n.f.I-max}$	${\delta A}_{ch-n.f.I-min}$	Θ_II_
type “P” (non-toothed)		0.399%	− 0.416%	0.00709
z_S_ = 9	type “S”	0.337%	− 0.630%	0.00831
	type “CH”	0.319%	− 0.392%	0.00616
z_S_ = 14		0.045%	− 0.971%	0.01096

**Figure 12:**
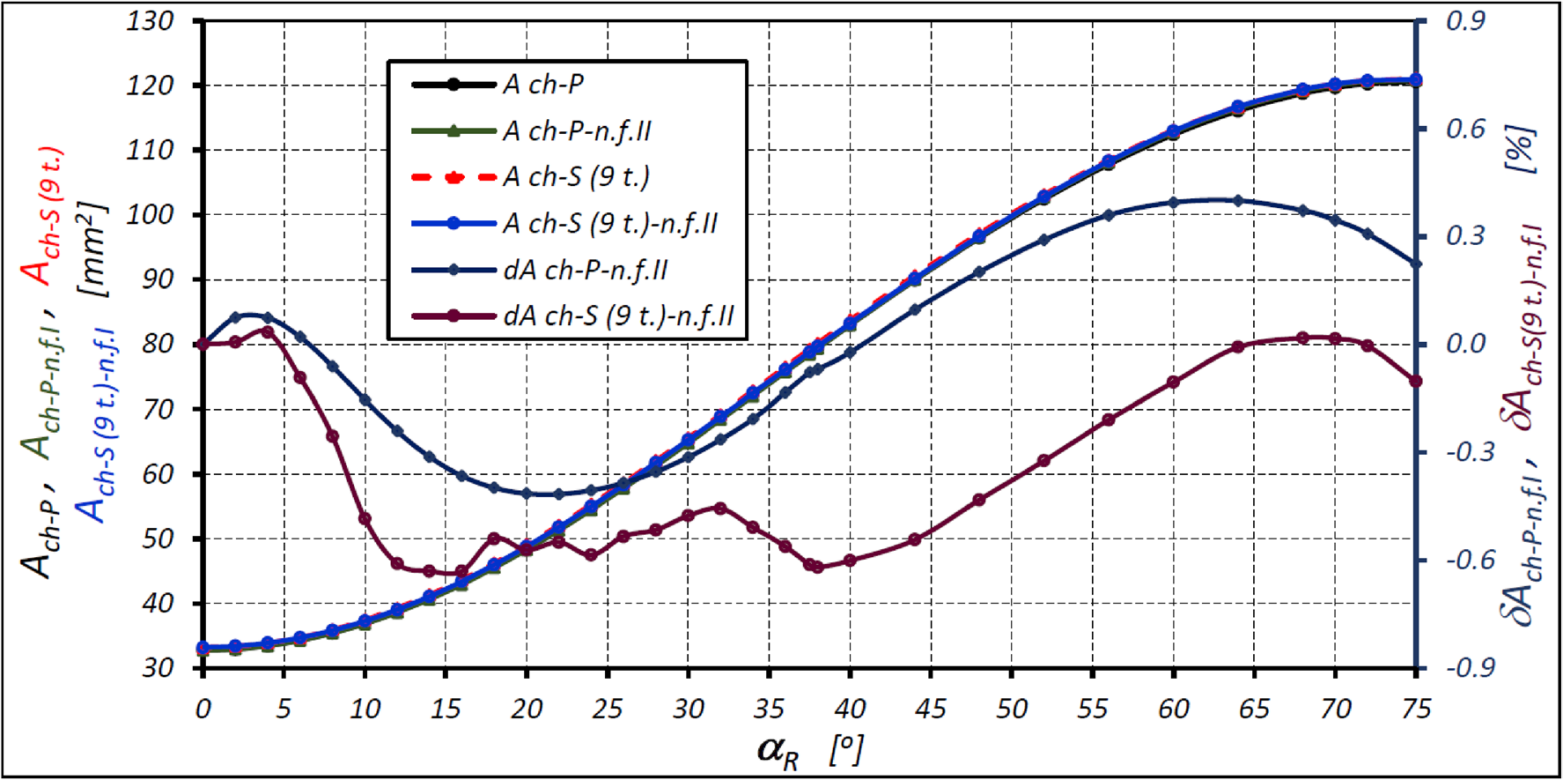
Areas A_ch_ of the working chamber of the satellite mechanism of variant II with nine-tooth satellite (z_S_ = 9) as a function of the angle α_R_ of the rotor rotation calculated according to formula (46) and the relative differences of the areas. Designations as in Fig. 10.

The characteristics of the areas calculated according to formula (46) for the satellite mechanism of variant II with the nine-tooth satellite are shown in Fig. 12.

The above characteristics show that the use of formula (46) means that the relative difference in the calculated area of the working chamber of the mechanism with a nine-tooth satellite is not more than 0.63%. A larger difference was determined for the mechanism with a fourteen-tooth satellite (0.65%—Table 7).

### Model III

The characteristics presented in Fig. 10 show that the greatest differences between the chamber areas calculated according to the known formula (39) and those determined from the CAD documentation occur in the middle range of the rotor rotation angle α_R_. Therefore, changes can be made to the cosine function in formula (39) to obtain the minimum difference in areas. The proposed new formula is as follows:

47 $${A}_{ch}=0.5\cdot \left({A}_{ch-max}-{A}_{ch-min}\right)\cdot \left[1-{\text{cos}}\left({\alpha }_{III}\right)\right]+{A}_{ch-min}$$

48 $${\alpha }_{III}={n}_{CR}\cdot \left({\alpha }_{R}+{\Theta }_{III}\cdot {\text{sin}}\left({\alpha }_{R}\cdot {\Theta }_{IV}\cdot {n}_{CR}\right)\right)$$

where Θ_III_ and Θ_IV_ are coefficients whose values are chosen so that conditions (43) and (44) are satisfied.

In Table 8, the values of the coefficients Θ_III_ and Θ_II IV_ and the relative differences in the working chamber area of the considered mechanism of variant II are given and calculated according to formula (45). The working chamber areas were calculated using the formula (47) and according to the CAD documentation (as in Fig. 7).

**Table 8:** The relative differences in % in the calculation of the chamber area of the satellite mechanism of variant II according to model III and the values of the coefficients Θ_III_ and Θ_II_.

Mechanism		${\delta A}_{ch-n.f.I-max}$	${\delta A}_{ch-n.f.I-min}$	Θ_III_	Θ_IV_
type “P” (non-toothed)		0.152%	− 0.152%	1.0765	1.205
z_S_ = 9	type “S”	0.200%	− 0.200%	1.2160	1.145
	type “CH”	0.161%	− 0.161%	1.0861	1.170
z_S_ = 14		0.196%	− 0.196%	1.1553	1.110

**Figure 13:**
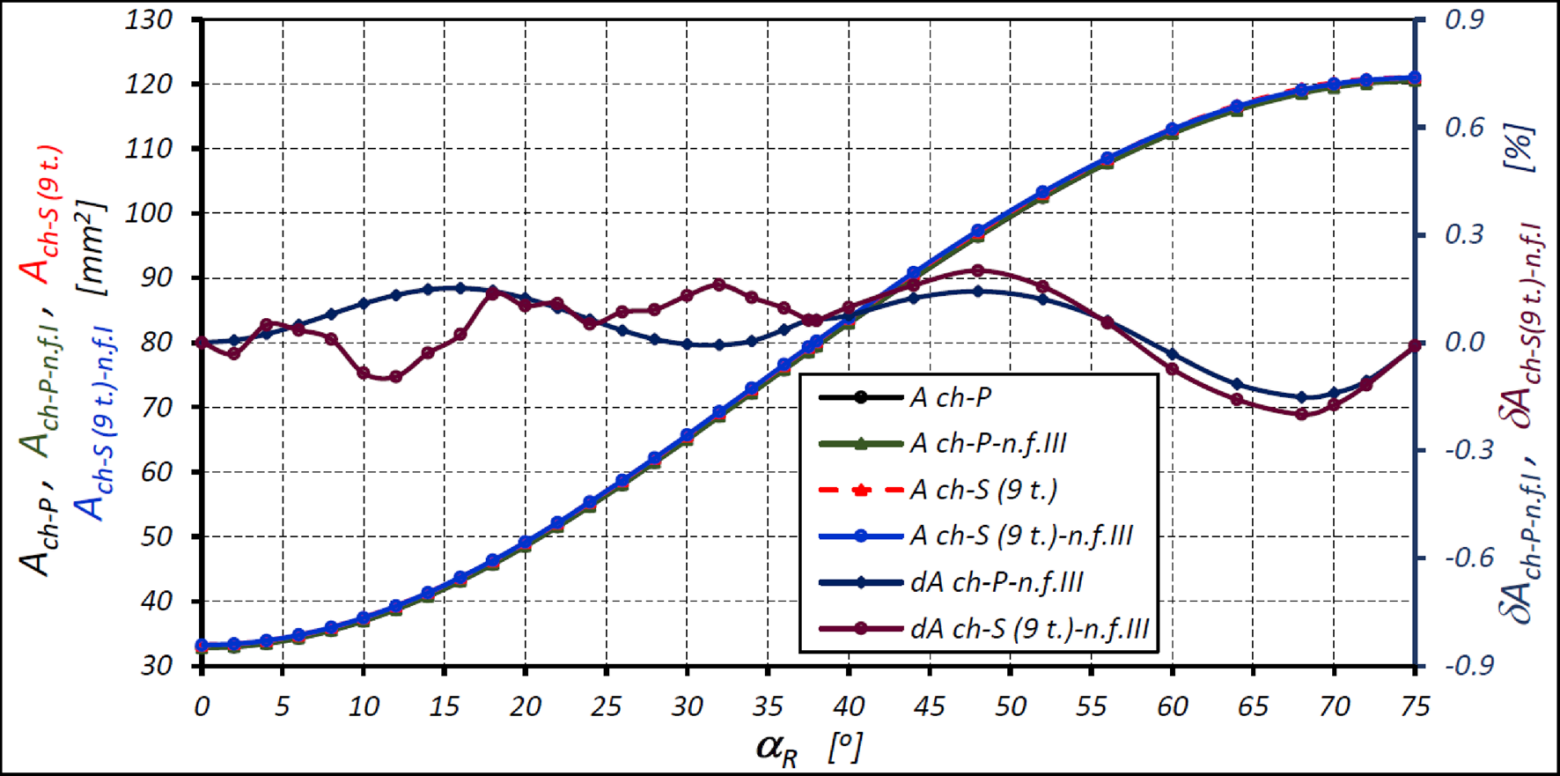
Areas A_ch_ of the working chamber of the satellite mechanism of variant II with nine-tooth satellite (z_S_ = 9) as a function of the angle α_R_ of the rotor rotation calculated according to formula (47) and the relative differences of the areas. Designations as in Fig. 10.

The characteristics of the areas calculated according to formula (47) for the satellite mechanism of variant II with the nine-tooth satellite are shown in Fig. 13.

The above characteristics show that the use of formula (47) means that the relative difference in the calculated area of the working chamber of the mechanism with a nine-tooth satellite is not more than 0.20% and is smaller that the difference obtained from model I. Similarly, the relative difference does not exceed 0.20% for all types of areas (Table 8).

#### Relative differences in the chamber area values of the toothed satellite mechanism for the coefficient values as for the non-toothed mechanism

Table 9 shows the values of the relative differences (calculated according to (45)) of the chamber area of the considered mechanism of variant II, calculated according to the models described above (formulas (39), (42), (46) and (47)) and the CAD documentation (as in Fig. 7) assuming the coefficients Θ_I_, Θ_II_, Θ_III_ and Θ_IV_ as for the non-toothed mechanism (type “P”).

**Table 9:** The relative differences in % in the calculation of the chamber area of the satellite mechanism of variant II according to all models and values of the coefficients of these models as for the non-toothed mechanism.

Model	Mechanism		Θ_I_	Θ_II_	Θ_III_	Θ_IV_	${\delta A}_{ch-n.f.I-max}$	${\delta A}_{ch-n.f.I-min}$
k.f	type “P” (non-toothed)		–	–	–		0	− 2.950%
	z_S_ = 9	type “S”						− 3.176%
		type “CH”						− 3.027%
	z_S_ = 14							− 3.266%
I	type “P” (non-toothed)		0.03408				0.728%	− 0.728%
	z_S_ = 9	type “S”					0.638%	− 0.954%
		type “CH”					0.618%	− 0.833%
	z_S_ = 14						0.778%	− 1.050%
II	type “P” (non-toothed)			0.00709			0.399%	− 0.416%
	z_S_ = 9	type “S”					0.072%	− 0.928%
		type “CH”					0.268%	− 0.596%
	z_S_ = 14						0.223%	− 1.291%
III	type “P” (non-toothed)		–	–	1.0765	1.205	0.152%	− 0.152%
	z_S_ = 9	type “S”					0.035%	− 0.317%
		type “CH”					0.170%	− 0.209%
	z_S_ = 14						0.198%	− 0.472%

The comparison of the results in the table above with the results in Table 6, Table 7 and Table 8 shows that the assumption the values of the coefficients of type Θ for the toothed mechanism as for the toothless mechanism leads to an underestimation of the value of the calculated chamber area.

## Conclusions

From the CAD documentation of the working mechanisms (Fig. 4) and the results contained in Table 3 and Table 5, it can be concluded that:

- the area of the minimum chamber of the toothed mechanism of variant I is greater than the area of the minimum chamber of the mechanism of variant II (A_ch-min-S-I_ = 1.456A_ch-min-S-II_);
- the area of the maximum chamber of the mechanism of variant II is smaller than the area of the maximum chamber of the mechanism variant II (A_ch-max-S-I_ = 0.895 A_ch-max-S-II_);
- the working volume of the mechanism of variant II is more than 46% larger than the working volume of the mechanism of variant I, because ΔA_ch-S-II_ = 1.465 ΔA_ch-S-I_. Therefore, the working mechanism of variant II should be used in hydraulic displacement machines (pumps and motors), as it has a favourable effect on reducing the specific mass of these machines;
- the A_ch-min_ and A_ch-max_ areas of the „S” type chamber (real toothed mechanism) differ from the areas of the "P" type chamber by up to 0.4%. Due to the fact that the toothed mechanism is used in positive displacement machines, the areas of this mechanism should be used to calculate the working volume (A_ch-min-S_ = 1.456A_ch-max-S_);
- in backlash-free mechanisms (the chisel is a satellite), a smaller value of both the minimum and the maximum area of the working chamber is obtained and compared to the mechanism with a classic satellite (Table 3). However, this difference is very small (less than 0.9—Table 5). Therefore, the use of backlash-free mechanisms is not justified. Another factor in favour of not using the backlash-free mechanism is the higher precision required in manufacturing and therefore the associated higher production cost;
- in the mechanism with a fourteen-tooth satellite, the area of both the maximum and the minimum working chambers is about 0.5% larger than the area of the chambers in the mechanism with a nine-tooth satellite (Table 3). It is therefore advisable to use a smaller number of teeth on the satellite, as a mechanism with greater tooth strength is obtained at the expense of a minimal loss of geometric working volume.

However, Fig. 9 shows that the area of the working chamber changes non-linearly from the A_ch-min_ to A_ch-max_ depending on the rotor rotation angle α_R_. To calculate the value of the chamber area A_ch_ for any rotor rotation angle α_R_, empirical formulas based on the trigonometric functions sine and cosine can be used. These are the formulas (39), (42), (46) and (47). The formula (39) is known in the literature and leads to lower calculation results for the chamber area, namely by more than 0.7% (Table 6 and Fig. 11). Lower difference values result from calculations according to the proposed formulas, i.e. formulas (42), (46) and (47). However, formula (47) provides the smallest deviations in the calculation results—no more than 0.2% (Table 8 and Fig. 13). Therefore, it is proposed to use it for the calculation of the area of the working chamber of the satellite positive displacement machine.

It is also noticeable that the number of teeth in the satellite mechanism and the tip clearances also influence its geometric working volume q_g_ (Table 1, Table 2, Table 3 and Fig. 6). Due to the fact that backlash-free mechanisms are not suitable for use in positive displacement machines, no further consideration is given to the working chamber areas (type "CH") of backlash-free mechanisms. However, the real mechanisms, i.e. mechanisms with areas of type "S", are not without significance. Reading the working chamber areas from the CAD documentation for different rotor rotation angles α_R_ is very time-consuming and sometimes problematic. It is less problematic to read the areas of the non-toothed chamber (“P” type areas). On the basis of these areas and the data (coefficients) contained in Sect. 5, the models developed above (formulae (42), (46) and (47)) can be made dependent on the number of teeth z_S_ of the satellite. Because model III (formula (47)) is the most accurate, considerations about models I and II are omitted. Therefore, it is proposed to write model III in the form:

49 $${A}_{ch}=0.5\cdot \left({A}_{ch-{\text{max}}(zs)}-{A}_{ch-{\text{min}}(zs)}\right)\cdot \left[1-{\text{cos}}\left({\alpha }_{III}\right)\right]+{A}_{ch-{\text{min}}(zs)}$$

where:

50 $${\alpha }_{III}={n}_{CR}\cdot \left({\alpha }_{R}+{\Theta }_{III(zs)}\cdot {\text{sin}}\left({\alpha }_{R}\cdot {\Theta }_{IV(zs)}\cdot {n}_{CR}\right)\right)$$

51 $${A}_{ch-{\text{min}}(zs)}={A}_{ch-P-min}+0.03896\cdot {z}_{S}+0.10836$$

52 $${A}_{ch-{\text{max}}(zs)}={A}_{ch-P-max}+0.11122\cdot {z}_{S}-0.51258$$

53 $${\Theta }_{III(zs)}={\Theta }_{III-P}-0.01214\cdot {z}_{S}+0.24876$$

54 $${\Theta }_{IV(zs)}={\Theta }_{IV-P}+0.253\cdot {z}_{S}-2.337$$

On the basis of the above formula (49) and in accordance with formula (24), the area A_ch_ of the working chamber can be calculated for:

- different modules of teeth of the mechanism;
- different number z_S_ of satellite teeth, provided that the shape of the pitch line of the mechanism elements is maintained (as in the case of the mechanism with a nine- and fourteen-teeth satellite considered above).

It is also possible to calculate on the basis of formula (49):

- the volume V_ch_ of a working chamber for each rotor rotation angle α_R_;
- the geometric working volume q_g_ of the entire satellite mechanism for each rotor rotation angle α_R_;
- the unevenness of the absorption of the satellite mechanism at constant rotor speed;
- the unevenness of the theoretical torque at a constant pressure difference in the working chambers of the mechanism.

The above topics will be the subject of separate publications.

## Data Availability

The datasets used and/or analysed during the current study are available from the corresponding author on reasonable request.
